# Pioglitazone Protects Tubular Epithelial Cells during Kidney Fibrosis by Attenuating miRNA Dysregulation and Autophagy Dysfunction Induced by TGF-β

**DOI:** 10.3390/ijms242115520

**Published:** 2023-10-24

**Authors:** Anna Manzéger, Gantsetseg Garmaa, Miklós M. Mózes, Georg Hansmann, Gábor Kökény

**Affiliations:** 1 Institute of Translational Medicine, Semmelweis University, Nagyvárad tér 4, 1089 Budapest, Hungary; anna.manzeger@gmail.com (A.M.); gantsetseg.garmaa@gmail.com (G.G.); mozesmiklos@yahoo.com (M.M.M.); 2 International Nephrology Research and Training Center, Semmelweis University, Nagyvárad tér 4, 1089 Budapest, Hungary; 3 Department of Pediatric Cardiology and Critical Care, Hannover Medical School, 30625 Hannover, Germany; georg.hansmann@gmail.com

**Keywords:** TGF-β, kidney fibrosis, transcription factors, PPARγ, miRNA, autophagy

## Abstract

Excessive renal TGF-β production and pro-fibrotic miRNAs are important drivers of kidney fibrosis that lack any efficient treatment. Dysfunctional autophagy might play an important role in the pathogenesis. We aimed to study the yet unknown effects of peroxisome proliferator-activated receptor-γ (PPARγ) agonist pioglitazone (Pio) on renal autophagy and miRNA dysregulation during fibrosis. Mouse primary tubular epithelial cells (PTEC) were isolated, pre-treated with 5 µM pioglitazone, and then stimulated with 10 ng/mL TGF-β1 for 24 h. Male 10-week-old C57Bl6 control (CTL) and TGF-β overexpressing mice were fed with regular chow (TGF) or Pio-containing chow (20 mg/kg/day) for 5 weeks (TGF + Pio). PTEC and kidneys were evaluated for mRNA and protein expression. In PTEC, pioglitazone attenuated (*p* < 0.05) the TGF-β-induced up-regulation of Col1a1 (1.4-fold), Tgfb1 (2.2-fold), Ctgf (1.5-fold), Egr2 (2.5-fold) mRNAs, miR-130a (1.6-fold), and miR-199a (1.5-fold), inhibited epithelial-to-mesenchymal transition, and rescued autophagy function. In TGF mice, pioglitazone greatly improved kidney fibrosis and related dysfunctional autophagy (increased LC3-II/I ratio and reduced SQSTM1 protein content (*p* < 0.05)). These were accompanied by 5-fold, 3-fold, 12-fold, and 2-fold suppression (*p* < 0.05) of renal Ccl2, Il6, C3, and Lgals3 mRNA expression, respectively. Our results implicate that pioglitazone counteracts multiple pro-fibrotic processes in the kidney, including autophagy dysfunction and miRNA dysregulation.

## 1. Introduction

Many chronic kidney diseases (such as diabetic and hypertensive nephropathy, FSGS, etc.) progress to fibrosis with complete loss of renal function (end-stage renal disease), representing a serious health burden [1]. Transforming growth factor-β_1_ (TGF-β_1_) plays a pivotal role in the molecular pathogenesis of tissue fibrosis [2,3]. TGF-β1 can directly induce collagen synthesis [4] and impair tight junctions in renal tubular epithelial cells, changing cell physiology and promoting epithelial-to-mesenchymal transition (EMT) [5,6]. The peroxisome proliferator-activated receptor-γ (PPARγ) is a vasoprotective nuclear hormone receptor and transcription factor that modulates cell metabolism and attenuates fibrotic processes. For instance, the PPARγ-agonist pioglitazone has been used in the treatment of diabetic patients [7] and to prevent macrovascular complications in patients with insulin resistance [8]. PPARγ agonists have also been reported to dampen kidney damage in several experimental models such as ischemia reperfusion injury, autosomal dominant polycystic kidney disease (ADPKD), and nondiabetic chronic kidney diseases [9].

Several transcription factors have been associated with fibrotic diseases. As such, early growth response factor-1 and -2 (EGR1, EGR2) were identified to promote kidney fibrosis in mice, rats, and human FSGS [10,11]. EGR1 can stimulate collagen synthesis [12] and induce autophagy-related LC3B expression in pulmonary disease [13]. Still, the role of EGR1 and EGR2 in renal autophagy remains obscure. Signal transducers and activators of transcription (STATs) also play an important role in renal diseases [14]. STAT3 can induce cell proliferation [15,16] and promote fibrosis via TGF-β1 [17,18,19]. STAT3 can also activate CCL2 (MCP-1), promoting inflammation that supports tumor growth in the liver [20]. CCL2 also plays a crucial role in renal tubulointerstitial inflammation [21]. Galectin-3 (Lgals3) is another factor in the inflammatory response. Up-regulated galectin-3 was found in the UUO mouse model of progressive renal fibrosis, and its absence confers protection against renal myofibroblast accumulation, activation, and fibrosis [22]. IL-6 can also stimulate proximal tubular epithelial cells to produce collagen I and accelerate tubulointerstitial fibrosis, correlated with increased STAT3 phosphorylation [23]. In addition, complement component C3 can also induce tubulointerstitial inflammation and fibrosis through the TGF-β1/CTGF signaling pathway [24,25]. However, the effects of pioglitazone on these inflammatory factors during TGF-β-induced kidney fibrosis remain unclear.

Numerous miRNAs have been identified in acute or chronic kidney diseases, such as miR-21-5p, which strongly correlates with fibrosis progression [26]. TGF-β also induces pro-fibrotic miR-130 in human renal proximal tubular epithelial cells [27] and miR-199a in the kidneys of hypertensive rats as well [28]. The miR-199a forms a pre-miR that undergoes RNA processing to yield miR-199a-5p and miR-199a-3p. Elevated renal miR-199a-3p can trigger STAT3 activation [29], while increased miR-199a-5p was reported in both murine and human CKD [30]. Nevertheless, the possible effect of PPARγ agonist treatment on the renal expression of the miR-130 or miR-199a family has not been investigated yet. 

Autophagy in eukaryotic cells is responsible for enzymatic degradation of damaged and unnecessary proteins and organelles that, among others, involves LC3 (ATG8) and SQSTM1 (p62) as well-established indicators of the process [31]. Accumulating evidence indicates that autophagy plays a critical role in kidney maintenance and diseases [31]. A pro-survival role of autophagy was demonstrated in renal proximal tubular cells [32,33] and in acute kidney injury [34,35]. Meanwhile, the role of autophagy in chronic kidney disease (CKD) yielded conflicting outcomes, as it was reported to both alleviate CKD progression [36] and to worsen kidney fibrosis [37]. Thus, the role of autophagy in the pathogenesis of renal fibrosis and its relationship to PPARγ remains obscure.

In the present study, we aimed to elucidate the in vitro and in vivo impact of pioglitazone treatment on renal tubular cell processes playing a crucial role in the TGFβ-induced fibrotic response, such as autophagy, the regulation of pro-fibrotic miR-130 and miR-199a, and inflammation. We report that five-week oral administration of pioglitazone effectively ameliorated the fibrosis-related dysfunctional autophagy in mouse renal tubular epithelial cells and reduced miR-130 and miR-199a expression as well as inflammatory response. Our results underline the beneficial effect of PPARγ in reversing multiple pro-fibrotic processes in the kidney.

## 2. Results

### 2.1. Pioglitazone Dampens the TGF-β-Induced Fibrotic Response in Tubular Epithelial Cells

**Figure 1:**
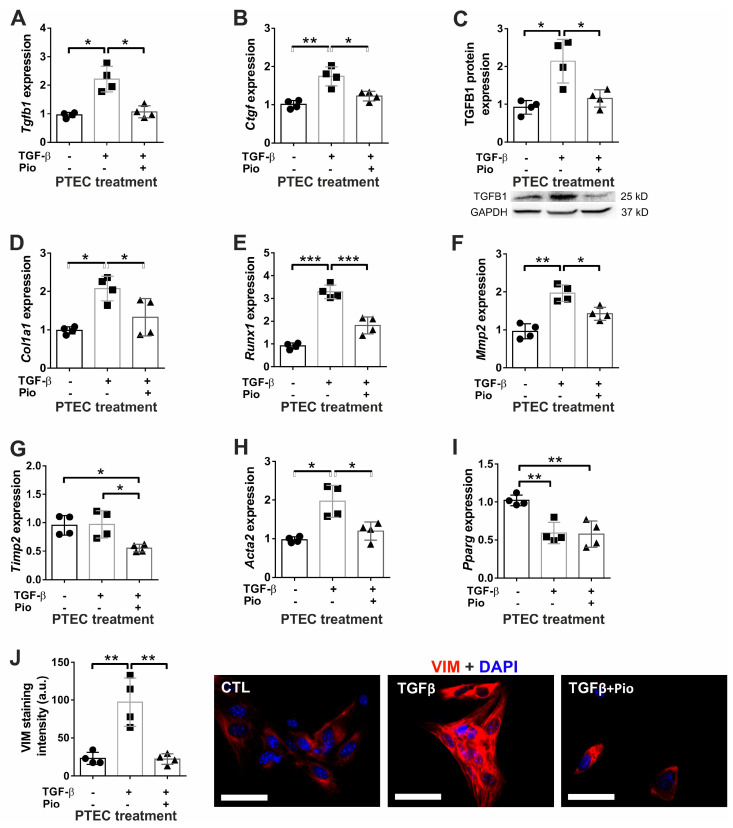
The effect of pioglitazone on the fibrotic response and EMT of murine primary tubular epithelial cells. TGF-β1 treatment for 24 h (10 ng/mL) increased, but 10 μM pioglitazone reduced the expression of (**A**) *Tgfb1* and (**B**) *Ctgf* mRNA, as well as (**C**) TGF-β1 protein (TGFB1) and (**D**) collagen-1 mRNA (*Col1a1*). (**E**) TGF-β1 induced the over-expression of *Runx1* (**E**) and *Mmp2* (**F**), which were abolished by pioglitazone. *Timp2* mRNA expression was not affected by TGF-β but reduced by pioglitazone (**G**). TGF-β1 also induced (**H**) α-SMA (*Acta2*) mRNA and (**J**) vimentin protein (VIM) which were abolished by pioglitazone pre-treatment. (**I**) PPARγ mRNA expression (*Pparg*) was reduced by 50% upon TGF-β1 treatment but unaffected by pioglitazone. Empty bars represent PTEC treatment groups (*n* = 4/group; TGFβ: 10 ng/mL; Pio: 5 μM pioglitazone). Target gene expressions were normalized to 18S rRNA. VIM staining intensity in arbitrary units (a.u.) was calculated using ImageJ v1.53 (VIM: red; nuclear stain DAPI: blue; 400× magnification, white scale bar represents 20 µm). Data are presented as mean ± SD. One-way ANOVA and Holm–Sidak post hoc test (SPSS 28 for Windows (IBM Inc, Armonk, NY, USA)), *: *p* < 0.05; **: *p* < 0.01; ***: *p* < 0.001.

**Figure 2:**
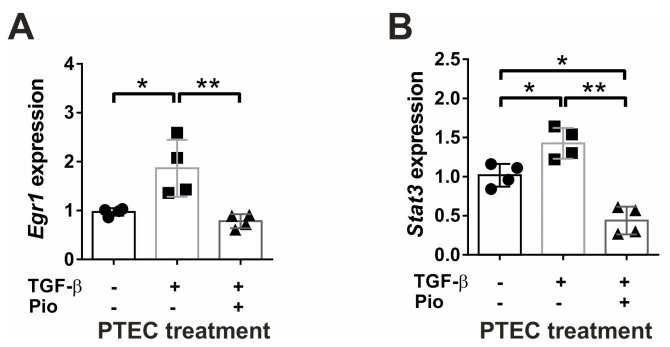
Pioglitazone inhibits TGF-β induced EGR1 and STAT3 transcription in PTEC cells. The mRNA expression of *Egr1* (**A**) and *Stat3* (**B**) were significantly induced by 24 h TGF-β treatment (10 ng/mL) but maintained at control levels by 10 μM pioglitazone (*n* = 4/group). Data are presented as mean ± SD. One-way ANOVA and Holm–Sidak post hoc test (SPSS 28 for Windows (IBM Inc., Armonk, NY, USA)), *: *p* < 0.05; **: *p* < 0.01.

We have previously demonstrated that pioglitazone in vivo reduced the TGF-β induced glomerulosclerosis and tubulointerstitial fibrosis [38]. Thus, we aimed to elucidate whether pioglitazone affects EMT and fibrogenesis in vitro using mouse primary renal tubular epithelial (PTEC) cells. TGF-β administration induced the up-regulation of pro-fibrotic *Tgfb1* and *Ctgf* mRNA and TGFB1 protein, accompanied by two-fold increase in *Col1a1* (Figure 1A–D), and all these inductions of pro-fibrotic gene expression were effectively inhibited by pioglitazone treatment. These data support our previous in vivo results [38]. Additionally, TGF-β up-regulated the pro-fibrotic *Runx1* transcription factor (Figure 1E) and the matrix degrading *Mmp2* (Figure 1F) that were significantly abolished by pioglitazone treatment. Although TGF-β did not increase the mRNA expression of the MMP2 inhibitor *Timp2* (Figure 1G), pioglitazone reduced its expression below control levels. TGF-β also induced the EMT process in PTEC as shown by elevated *Acta2* mRNA and vimentin protein expression (Figure 1H,J), while pioglitazone decreased expression of both of these transcripts to control levels. Interestingly, the nearly 50% reduction in *Pparg* expression upon TGFβ administration was not influenced by pioglitazone treatment in vitro (Figure 1I). Additionally, we also observed the beneficial effect of pioglitazone treatment on *Egr1* and *Stat3* mRNA expression in PTEC cells (Figure 2A,B), supporting our previous in vivo results [35].

**Figure 3:**
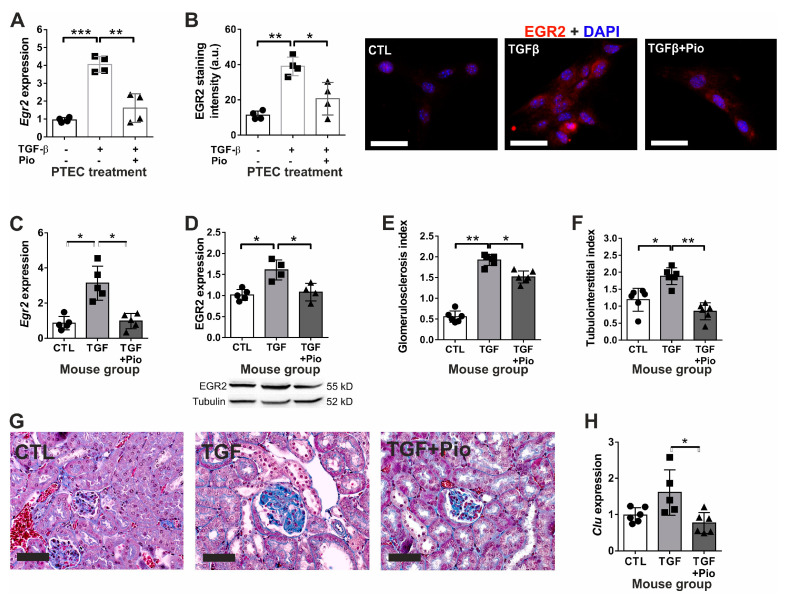
The effect of TGF-β and pioglitazone on the renal pro-fibrotic EGR2 response in vitro and in vivo. In murine PTEC cells, TGF-β induced EGR2 mRNA (*Egr2*) (**A**) and protein (**B**) expression with nuclear EGR2 shuttling, and all these effects were reduced by pioglitazone. The increased EGR2 mRNA (**C**) and protein (**D**) expressions in TGF-β overexpressing mouse kidneys (TGF) were accompanied by (**E**) marked glomerulosclerosis and (**F**) tubular cell injury with (**G**) dilatation and epithelial cell desquamation and (**H**) mild up-regulation of clusterin mRNA (*Clu*), all alleviated in pioglitazone treated-mice (TGF + Pio). Empty bars represent PTEC treatment groups (*n* = 4/group; TGF-β: 10 ng/mL; Pio: 5 μM pioglitazone). Filled bars represent mouse groups (*n* = 6/group): CTL: wild-type controls, TGF: un-treated TGF-β transgenic mice; TGF + Pio: pioglitazone-treated (20 mg/kg/day) TGF-β transgenic mice. Target gene expressions were normalized to 18S rRNA. EGR2 staining intensity in arbitrary units (a.u.) was calculated using ImageJ v1.53 (EGR2: red; nuclear stain DAPI: blue; co-localization: purple; 400× magnification, white scale bar represents 20 µm). Representative photomicrographs of Masson’s trichrome stained mouse kidneys were taken at 400× magnification (black scale bar represents 50 µm). Data are presented as mean ± SD. One-way ANOVA and Holm–Sidak post hoc test (SPSS 28 for Windows (IBM Inc., Armonk, NY, USA)), *: *p* < 0.05; **: *p* < 0.01; ***: *p* < 0.001.

EGR2 plays a pivotal role in renal fibrogenesis [10]; therefore, we wished to investigate whether pioglitazone has any effect on TGF-β-induced Egr2 expression both in vitro and in vivo. Mouse PTEC cells treated with TGF-β1 depicted a four-fold *Egr2* up-regulation that was prevented by pioglitazone (Figure 3A). EGR2 immunostaining of the cells depicted barely detectable levels in untreated controls but over-expression upon TGF-β treatment with significant nuclear localization, while pioglitazone treatment dampened the TGF-β-induced EGR2 up-regulation (Figure 3B). 

**Figure 4:**
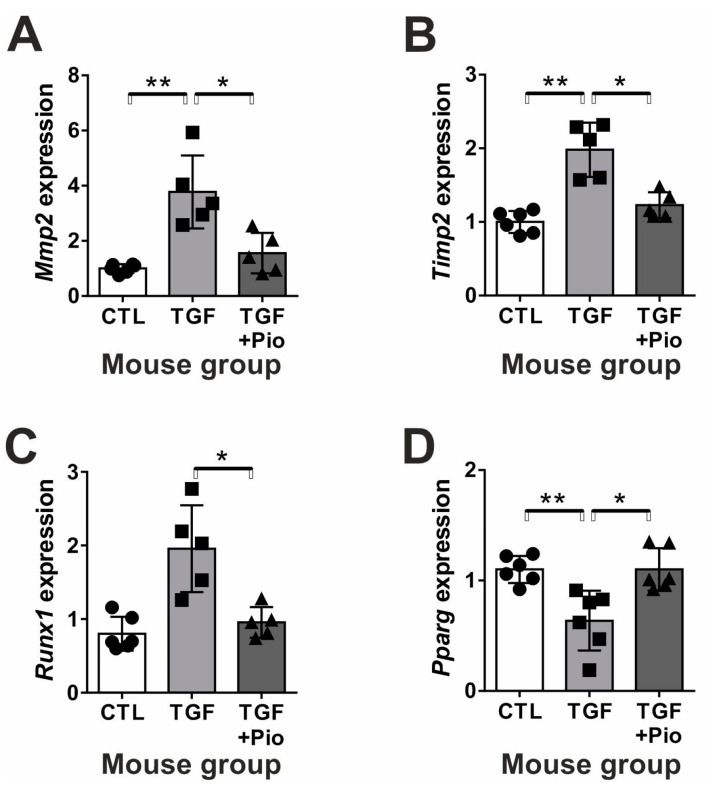
Pioglitazone reverses the dysregulated renal gene expression of fibrosis-related factors in TGF-β transgenic mice. The renal mRNA expression of (**A**) matrix metalloproteinase-2 (*Mmp2*) and its tissue inhibitor (**B**) TIMP-2 (*Timp2*) as well as (**C**) runt-related transcription factor-1 (*Runx1*) were induced by TGF-β, but PPARγ (*Pparg*) (**D**) was reduced. Pioglitazone reversed all these expression changes to control levels. Filled bars represent mouse groups (*n* = 6/group): CTL: wild-type controls, TGF: un-treated TGF-β transgenic mice; TGF + Pio: pioglitazone-treated (20 mg/kg/day) TGF-β transgenic mice. Target gene expressions were normalized to 18S rRNA. Data are presented as mean ± SD. One-way ANOVA and Holm–Sidak post hoc test (SPSS 28 for Windows (IBM Inc., Armonk, NY, USA)), *: *p* < 0.05; **: *p* < 0.01.

We observed similar protective effects of chronic pioglitazone treatment in vivo. Untreated TGF-β transgenic mice exerted a significant *Egr2* mRNA (Figure 3C) and protein over-expression as compared to wild-type controls (Figure 3D), which were significantly reduced to control levels by the pioglitazone treatment. Pioglitazone inhibited further fibrotic responses in mouse kidneys. The extent of TGF-β induced glomerulosclerosis (Figure 3E,G) was mildly but significantly reduced by pioglitazone. However, tubulointerstitial damage (Figure 3F,G) induced by TGF-β was completely reversed by chronic pioglitazone treatment. Accordingly, clusterin (*Clu*) gene expression (as a marker of tubular damage) was significantly up-regulated in non-treated transgenic mice but remained at control level in pioglitazone-treated mice (Figure 3H). Similar to the results obtained on PTEC cells, the induced *Mmp2* and *Timp2* gene expressions in TGF-β transgenic mice were completely abolished by pioglitazone treatment, which also inhibited the up-regulation of *Runx1* transcription factor (Figure 4A–C). In contrast to PTEC cells, pioglitazone treatment in vivo practically normalized the TGF-β-induced massive reduction of renal *Pparg* expression (Figure 4D).

### 2.2. Pioglitazone Restores the Expression of TGF-β-Induced “fibromiRs”

**Figure 5:**
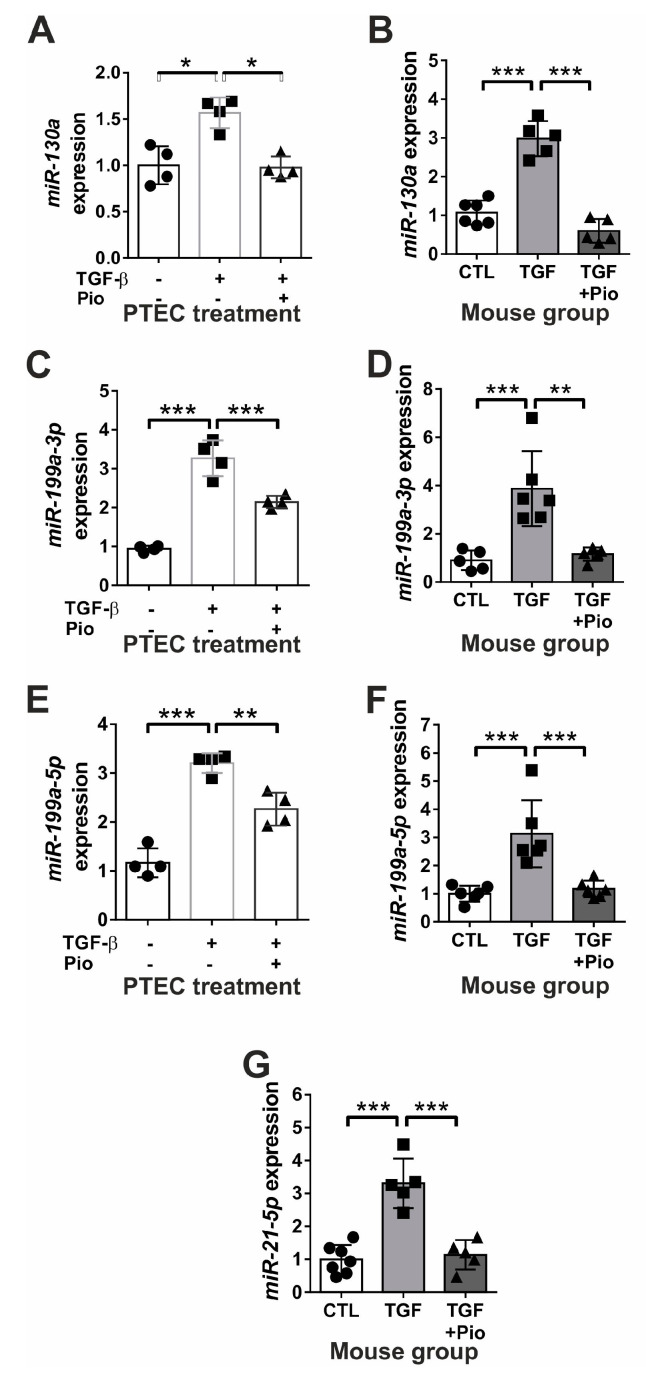
Pioglitazone attenuates the TGF-β induced dysregulation of pro-fibrotic miRNAs in vitro and in vivo. TGF-β induced but pioglitazone abolished the pro-fibrotic miR-130a (**A**) and both miR-199a-3p (**C**) and miR-199a-5p (**E**) over-expression in mouse PTEC cells. Similar miR-130a (**B**) and miR-199a (**D**,**F**) dysregulation accompanied by induced miR-21 (**G**) were observed in TGF-β-overexpressing mouse kidneys, attenuated by pioglitazone treatment. Empty bars represent PTEC treatment groups (*n* = 4/group; TGFβ: 10 ng/mL; Pio: 5 μM pioglitazone). Filled bars represent mouse groups (*n* = 6/group): CTL: wild-type controls, TGF: untreated TGF-β transgenic mice; TGF + Pio: pioglitazone-treated (20 mg/kg/day) TGF-β transgenic mice. Target miRNA expressions were normalized to U6 snRNA. Data are presented as mean ± SD. One-way ANOVA and Holm–Sidak post hoc test (SPSS 28 for Windows (IBM Inc, Armonk, NY, USA)), *: *p* < 0.05; **: *p* < 0.01; ***: *p* < 0.001.

In accordance with earlier observations on human kidney cells [27], our experiments confirmed that miR-130a expression was significantly up-regulated in our murine PTEC cells following TGF-β treatment (Figure 5A) and increased also in the kidneys of TGF-β transgenic mice (Figure 5B), but this up-regulation was effectively dampened by pioglitazone treatment. The significantly over-expressed renal miR-21-5p in TGF-β transgenic mice was normalized to control level after five-week oral pioglitazone administration (Figure 5G), providing further support for the in vivo pioglitazone treatment against the pro-fibrotic effects of TGF-β. 

Both miR-199a-3p and miR199a-5p are potential regulatory factors of TGF-β-mediated pathological renal processes [28,29,30]. TGF-β administration markedly increased the expressions of both miR-199a-3p and miR-199a-5p in primary mouse tubular epithelial cells, which were inhibited by pioglitazone (Figure 5C,E). Similar to these in vitro results, kidneys of untreated TGFβ transgenic mice depicted four-fold and three-fold miR-199a-3p and miR-199a-5p overexpression as compared to control kidneys, respectively, which were effectively reduced to the level of control kidneys by chronic pioglitazone treatment (Figure 5D,F).

### 2.3. Pioglitazone Protects Renal Tubular Cells during Fibrotic Processes by Ameliorating Autophagy Dysfunction

**Figure 6:**
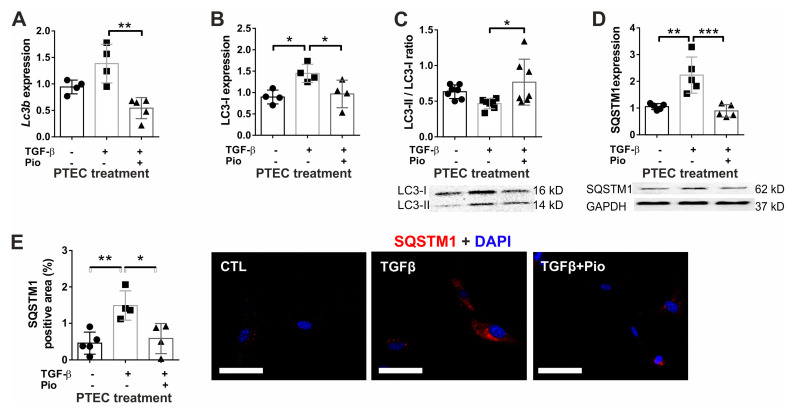
Pioglitazone protects tubular epithelial cells from TGF-β-induced autophagy dysfunction in vitro. In mouse PTEC cells, TGF-β administration induced autophagy with up-regulated LC3 mRNA (*Lc3b*) (**A**) and protein (LC3-I) (**B**). The reduced LC3-II/LC3-I ratio (**C**) with SQSTM1 (p62) protein accumulation (**D**,**E**) implied less autophagosome degradation, normalized by pioglitazone. Empty bars represent PTEC treatment groups (*n* = 4/group; TGF-β: 10 ng/mL; Pio: 5 μM pioglitazone). Target gene expressions were normalized to 18S rRNA. SQSTM1 staining was evaluated as percent of positive cytoplasmic area using ImageJ v1.53 (SQSTM1: red; nuclear stain DAPI: blue 400× magnification, white scale bar represents 20 µm). Data are presented as mean ± SD. One-way ANOVA and Holm–Sidak post hoc test (SPSS 28 for Windows (IBM Inc., Armonk, NY, USA)), *: *p* < 0.05; **: *p* < 0.01; ***: *p* < 0.001.

In order to assess the effects of pioglitazone on renal tubular autophagy dysfunction both in vitro and in vivo, LC3 and SQSTM1 (p62) were investigated. LC3-I is the cytosolic form and LC3-II associates with autophagosome membranes. Thus, an increased LC3-II/I ratio indicates induced autophagy or impaired degradation, leading to the accumulation of autophagosomes with un-degraded contents [31]. SQSTM1 is the most extensively studied adapter protein that undergoes autophagic degradation, thus its decreased protein levels signify a more intense autophagic flux [31]. In primary tubular epithelial kidney cells (TEC) exposed to human recombinant TGF-β, LC3 expression was up-regulated (Figure 6A,B). Nevertheless, despite this increase, autophagy degradation was not induced, as indicated by the reduced LC3-II/I ratio (Figure 6C) accompanied by SQSTM1 accumulation (Figure 6D). These findings were further supported by immunostaining that showed a significant SQSTM1 protein accumulation in TGF-β-treated cells that disappeared with pioglitazone treatment (Figure 6E).

**Figure 7:**
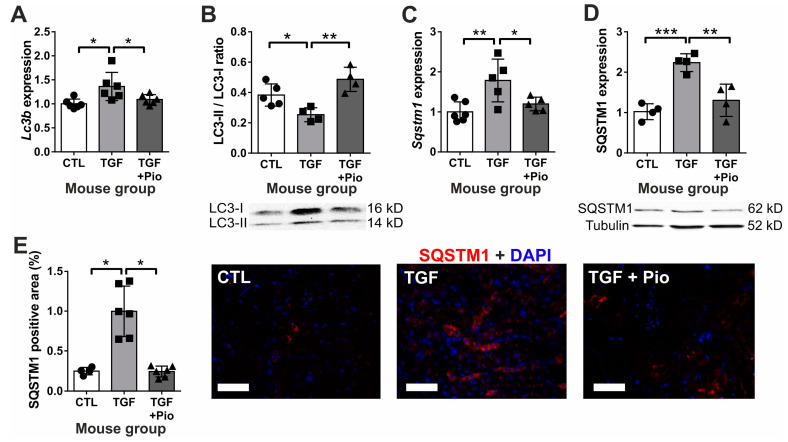
Pioglitazone reversed renal autophagy dysfunction induced by TGF-β in mice. TGF-β up-regulated renal *Lc3b* (**A**) expression. The reduced LC3-II/LC3-I protein ratio (**B**) with induced SQSTM1 mRNA (**C**) and protein expression (**D**) and SQSTM1 accumulation in kidney tubules (**E**) indicated autophagy dysfunction, which was restored by pioglitazone. Filled bars represent mouse groups (*n* = 6/group): CTL: wild-type controls, TGF: un-treated TGF-β transgenic mice; TGF + Pio: pioglitazone-treated (20 mg/kg/day) TGF-β transgenic mice. Target gene expressions were normalized to 18S rRNA. SQSTM1 staining was evaluated as percent of positive tubular area using ImageJ v1.53 (SQSTM1: red; nuclear stain DAPI: blue 400× magnification, white scale bar represents 50 µm). Data are presented as mean ± SD. One-way ANOVA and Holm–Sidak post hoc test (SPSS 28 for Windows (IBM Inc, Armonk, NY, USA)), *: *p* < 0.05; **: *p* < 0.01; ***: *p* < 0.001.

Similarly, the kidneys of TGF-β transgenic mice exhibited elevated LC3b and Sqstm1 expression levels. This was accompanied by a reduced LC3-II/I ratio and a noticeable accumulation of SQSTM1 (p62) (Figure 7A–E), suggesting that the up-regulation of gene expression did not translate into an increased rate of autophagy degradation. In contrast, chronic pioglitazone treatment ameliorated the TGF-β-induced autophagy dysfunction, as it improved the LC3-II/I ratio and reduced the accumulation of SQSTM1. Notably, SQSTM1 was mainly localized to the renal tubular epithelium (Figure 7E), supporting our in vitro results.

### 2.4. Pioglitazone Protects Renal Tubular Cells against TGF-β-Induced Inflammation

**Figure 8:**
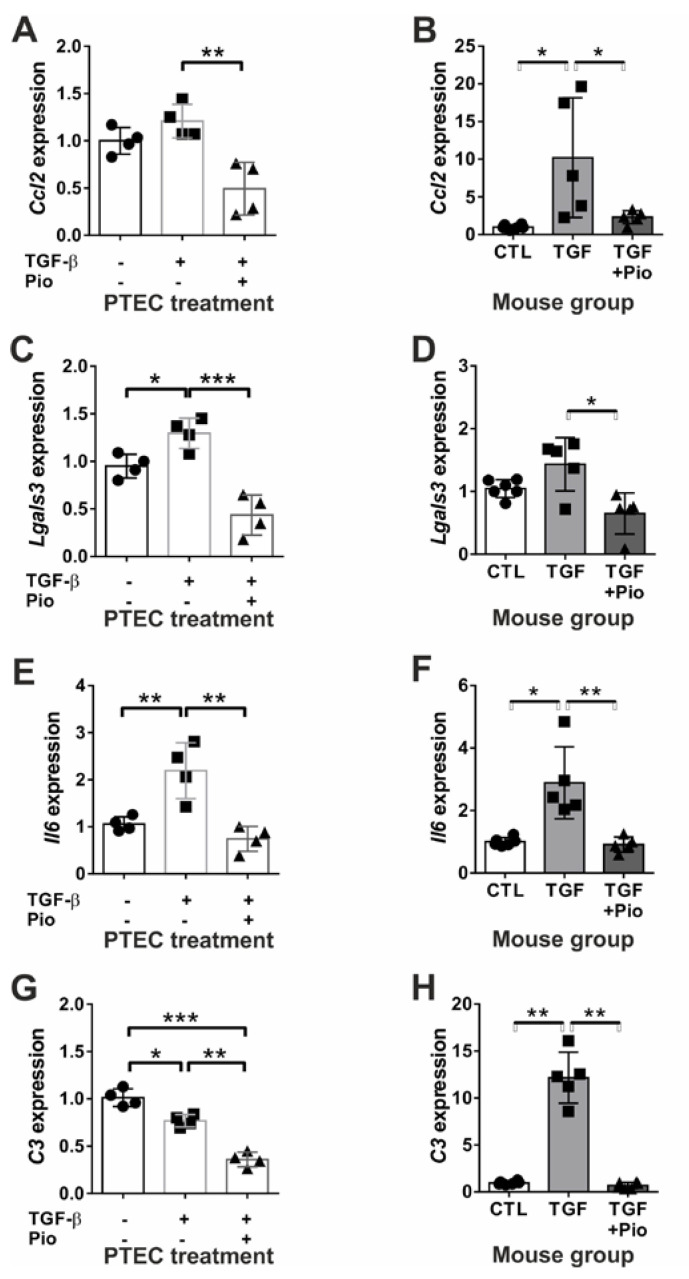
Pioglitazone inhibited the TGF-β-induced inflammatory response in vitro and in vivo. In mouse PTEC cells, TGF-β administration induced (**A**) MCP-1/CCL2 (*Ccl2*), (**C**) galectin-3 (*Lgals3*), and (**E**) *Il6* mRNA over-expression, which were all reduced to control levels by pioglitazone. (**G**) *C3* mRNA expression was suppressed by TGF-β and even further reduced by pioglitazone. TGF-β induced renal (**B**) *Ccl2*, (**D**) *Lgals3*, (**F**) *Il6*, and (**H**) *C3* mRNA expression, which were alleviated by pioglitazone treatment. Empty bars represent PTEC treatment groups (*n* = 4/group; TGF-β: 10 ng/mL; Pio: 5 μM pioglitazone). Filled bars represent mouse groups (*n* = 6/group): CTL: wild-type controls, TGF: untreated TGF-β transgenic mice; TGF + Pio: pioglitazone-treated (20 mg/kg/day) TGF-β transgenic mice. Target gene expressions were normalized to 18S rRNA. Data are presented as mean ± SD. One-way ANOVA and Holm–Sidak post hoc test (SPSS 28 for Windows (IBM Inc., Armonk, NY, USA)), *: *p* < 0.05; **: *p* < 0.01; ***: *p* < 0.001.

In primary tubular epithelial cells, the TGF-β-induced expression of *Ccl2* significantly decreased upon pioglitazone treatment (Figure 8A). Kidneys of TGF-β transgenic mice depicted similar results. As compared to the control group, these untreated TGFb mice had increased renal *Ccl2* mRNA expression that was reduced by 50% upon pioglitazone treatment (Figure 8B). We also observed a significant increase in *Lgals3* expression upon TGF-β treatment in primary tubular epithelial cells, but this effect was effectively antagonized by pioglitazone (Figure 8C). Similarly, pioglitazone significantly reduced the renal *Lgals3* expression in TGF-β-overexpressing mice (Figure 8D). The expression level of pro-inflammatory cytokine *Il6* showed a similar pattern in response to TGF-β and pioglitazone treatments both in vitro and in vivo (Figure 8E,F).

As local renal C3 may contribute to tubulointerstitial inflammation and fibrosis, we also examined whether C3 could be regulated by TGF-β and pioglitazone in PTEC and in mouse kidneys. Although renal *C3* expression was elevated in TGF-β transgenic mice (Figure 8H), this change is unlikely to be attributed to tubular cell alterations, as they rather showed a decreasing tendency in vitro (Figure 8G). However, pioglitazone treatment resulted in a significant reduction in *C3* expression both in the kidneys and in the primary tubular epithelial cells (Figure 8G,H).

## 3. Discussion

In the present study, we demonstrate that the PPARγ agonist pioglitazone protects renal tubular cells from TGF-β-induced pro-fibrotic response both in vitro and in vivo by ameliorating autophagy dysfunction, reducing pro-fibrotic miRNAs and EGR2, and inhibiting inflammation. To our knowledge, this is the first study showing that pioglitazone prevents the significant increase in EGR2 expression induced by TGF-β1 in both mouse PTEC cells and in TGF-β transgenic mouse kidneys, associated with reduced nuclear localization (shuttling) of EGR2.

While the early growth response-2 (EGR2) protein shares structural and functional similarities with EGR1, these two closely related early-immediate transcription factors serve partially distinct functions and are not redundant. EGR2 is critically involved in processes such as peripheral nerve myelination, adipogenesis, and immune tolerance [39]. Nevertheless, its involvement in kidney fibrosis has been only recently discovered [10]. TGF-β triggers rapid and transient up-regulation of EGR1 that, in response, stimulates EGR2, while both EGR1 and EGR2 directly enhance collagen gene expression [39]. The overexpression of EGR2 in vitro induces, whereas both in vitro and in vivo EGR2 silencing inhibits the TGF-β1-induced fibrotic response in the kidney [10]. In addition, EGR1 not only enhances renal collagen transcription [12], but it was also reported to induce autophagy-related LC3B expression in pulmonary disease [13]. 

In the kidney, proximal tubular epithelial cells exert relatively low levels of autophagy that can be induced by TGF-β [40]. Mature TGF-β is degraded by the autophagic pathway under TGF-β stimulation and auto-induction [36]. Defective autophagy might worsen renal fibrosis. For instance, the administration of autophagy inhibitor 3-MA enhanced tubular apoptosis and interstitial fibrosis in a rat unilateral ureter obstruction (UUO) model [36,41]. The heterozygous deletion of autophagic protein beclin-1 in mice increased type-1 collagen deposition in the kidneys [40]. Additionally, LC3-deficient and beclin-1-deficient mice subjected to UUO exhibited increased collagen deposition and TGF-β1 protein levels [36]. Our experiments indicate that—despite the transcriptional upregulation of autophagy related genes—tubular SQSTM1 protein levels remained elevated. This implicates dysfunctional degradation via autophagy upon TGF-β induction in both primary tubular epithelial cells and in the kidneys of TGF-β transgenic mice.

Various studies have confirmed the association between PPAR-γ and autophagy-related pathways. In bronchial epithelial cells, PPARγ agonists were found to increase the levels of autophagy-related proteins, including beclin-1 and LC3-II/I, while simultaneously decreasing the accumulation of SQSTM1, suggesting that PPARγ can promote autophagy [42]. This corroborates our observations, as the clinically approved PPARγ agonist and anti-diabetic drug pioglitazone effectively reduced the tubular accumulation of SQSTM1 and rescued the TGF-β-induced autophagy dysfunction both in vitro and in vivo.

Some miRNAs with abnormal expression have been also associated with fibrosis progression (referred to as fibromiRs) and were postulated as being potential therapeutic targets. For instance, the inhibition of miR-130a-3p protected against renal fibrosis in vitro through the TGF-β1/Smad pathway [27]. We have previously demonstrated that TGF-β1 decreases PPARγ mRNA via induction of the miR-130a/301b cluster in human and murine pulmonary artery SMC [19]. Additionally, PPARγ inhibits miR-130a expression in cardiac cells and pioglitazone mitigates doxorubicin-induced cardiotoxicity via miR-130a [43]. Corroborating these studies, we observed in mice with TGF-β-induced kidney fibrosis that five-week treatment with the PPARγ agonist pioglitazone reduced miR-130a expression to control levels. In addition, as previously demonstrated in HK2 cells [44], pioglitazone’s ability to reduce miR-21 expression was confirmed in our mouse kidneys as well. 

The miR-199a-5p and -3p can be generated from miR-199a-1 and -2 precursors and both can influence the TGF-β pathway. For instance, miR-199a-5p was reported to inhibit endoplasmic reticulum stress in renal ischemia-reperfusion (I/R) injury [45] and to down-regulate PPARγ, exacerbating renal interstitial fibrosis in rats with hyperuricemia [28]. Elevated miR-199a-5p has been reported in mouse and human CKD as well, underscoring its potential importance [30]. On the other hand, PPARγ might regulate the expression of miR-199a-5p by influencing the processing or stability of pre-miR-199a at the post-transcriptional level [46]. Other studies suggested miR-199a-3p to play a role in fibrosis development through the regulation of TGF-β signaling. Yang and colleagues showed that p53 increased renal miR-199a-3p expression, leading to STAT3 activation in UUO mice [29]. In rats, subtotal nephrectomy induced renal miR-199a-3p over-expression [47]. In renal tubular epithelial cells, miR-199a-3p expression was significantly elevated upon TGF-β1 treatment, and overexpression of miR-199a-3p influenced collagen synthesis [48]. Our experiments revealed that both miR-199a-5p and miR-199a-3p similarly increased in TGF-β-treated primary tubular epithelial cells and the kidneys of TGF-β-overexpressing mice, but the PPARγ agonist pioglitazone alleviated the dysregulation of miR-199a-3p and -5p.

The pathogenesis of kidney fibrosis can also be initiated or worsened by inflammation through multiple pathways, regulated by a complex interaction of different factors involving cytokines, chemokines, and adhesion molecules. CCL2 levels in human CKD patients correlated significantly with the progression of interstitial fibrosis [49]. Suppression of IL-6 in tubular epithelial cells hampered interstitial fibrosis and tubular atrophy. Conversely, chronic administration of IL-6 enhanced the fibrotic process [23]. Extracellular galectin-3 can modulate important interactions between epithelial cells and the extracellular matrix [50], and the absence of galectin-3 protects against renal myofibroblast accumulation and activation in the UUO kidney fibrosis model [22]. Additionally, studies suggest that functionally active C3 proteins and C3aR are expressed in proximal tubular epithelial cells and play an important role in the pathogenesis of kidney fibrosis [51,52]. In our study, fibrotic kidneys and TGF-β-treated tubular epithelial cells exerted up-regulation of pro-inflammatory transcripts *Il6*, *Ccl2*, and *Lgals3*, all of which were successfully attenuated by pioglitazone treatment. Our results are in accordance with previous studies reporting the anti-inflammatory actions of pioglitazone in coronary arteriosclerosis by reducing the expression of CCL2 receptor (CCR2) [53] as well as in traumatic brain injury by modulating the PPARγ/NF-κB/IL-6 pathway [54]. As part of its anti-inflammatory effect, pioglitazone attenuated the renal C3 over-expression in our TGF-β transgenic mice. Moreover, short-term pioglitazone administration exerted anti-inflammatory effects in diabetic nephropathy patients as well [55].

## 4. Materials and Methods

### 4.1. Primary Tubular Epithelial Cell Isolation and Cell Culture

A 4-week-old C57B1/6J male mouse was euthanized and both kidneys were quickly removed after median laparotomy under aseptic conditions. The kidneys were placed on a 10 cm cell culture dish containing an ice-cold sterile HBSS buffer and placed under a laminar flow box. The kidneys were de-capsulated and cut in several slices, and the medulla was excised. Chopped small cortical fragments were digested in Dulbecco’s Modified Eagle Medium:Nutrient Mixture F-12 (DMEM-F12) containing 1 mg/mL collagenase type-II for 20 min at 37 °C with vortexing every 10 min. The digested cortex was washed through a series of brass sieves with 100-60-40 µm mesh diameters. Tubules were collected from the 40 µm nylon mesh. The collected cells were resuspended in selective PTEC medium (DMEM-F12 containing 2% fetal bovine serum (FBS) (Gibco, Thermo Fisher Scientific, Waltham, MA USA), Insulin-Transferrin-Selenium-Sodium Pyruvate (ITS-A, Gibco), 40 ng/mL hydrocortisone (Sigma, Merck Life Science, Budapest, Hungary), 5 pg/mL triiodo-L-thyronine (Sigma), 50 U/mL penicillin and 50 μg/mL streptomycin (Sigma). The isolated mouse primary tubular epithelial cells (PTEC) were cultured in tissue culture flasks coated with 1% gelatin in a humidified atmosphere with 5% CO_2_ at 37 °C for 14 days. The purity of PTEC cultures was then validated by immunoblot and immunocytochemistry. Only the PTEC culture expressing epithelial marker E-cadherin without mesenchymal markers vimentin and α-SMA was passed into a new flask and used for the consecutive experiments.

PTEC cells with passage numbers P6 to P8 were seeded at a density of 1 × 10^5^ cells or 3 × 10^4^ cells per well on gelatin-coated 6-well and 24-well plates, respectively. On the following day, the medium was changed to serum-free PTEC medium, and the cells were subjected to pre-treatment with 5 µM pioglitazone (in 0.1% DMSO) or DMSO alone (controls). After 24 h of serum starvation, recombinant human TGF-β1 (10 ng/mL, Sigma-Aldrich) was added to the respective wells and incubated for an additional 24 h (*n* = 3–4/group).

### 4.2. Animal Experiments

Ten-week old male TGF-β1 transgenic mice on C57Bl6/J genetic background (B6-Alb/TGF-β1(cys^223,225^ser), originally obtained from Snorri S. Thorgeirsson at NCI, USA and backcrossed in our laboratory) [10,56] and wild-type C57Bl6/J males of the same age (controls, CTL, *n* = 6) were kept in a standard specific pathogen-free environment at the Semmelweis University NET GMO Animal Facility (Budapest, Hungary). Due to the hepatic expression of full-length active TGF-β1, transgenic mice exhibit elevated plasma TGF-β1 levels that result in progressive kidney fibrosis [56]. All the mice were maintained under a 10/14-h light/dark cycle and provided ad libitum access to rodent chow and drinking water. The presence of the transgene was verified from tail tip samples as previously described [7]. Blood sampling and euthanasia were performed under isoflurane anesthesia. All animal procedures were conducted with prior approval from the Semmelweis University Ethical Committee for Animal Welfare (PE/EA/948-4/2018). Transgenic mice were divided into two groups; one group received standard rodent chow (TGF, *n* = 6), the other group received rodent chow containing pioglitazone (TGF + Pio, *n* = 6) for chronic treatment (20 mg/kg/day, adjusted to the average food consumption). The mice were euthanized after 6 weeks of the treatment period with 5% isoflurane and perfused by intra-cardiac cannula with 4 °C physiological saline until blood was washed out of the kidneys. The kidneys were collected and cut to be frozen in liquid nitrogen or fixed in 4% buffered formalin solution.

### 4.3. Renal Histology and Immunohistochemistry

Masson’s trichrome staining was performed on formalin-fixed, paraffin-embedded kidney sections. The degree of glomerulosclerosis and tubulointerstitial damage was determined in a blinded fashion using a semi-quantitative scale as described previously [10]. 

Briefly, from the arithmetic mean of 100 evaluated glomeruli, the glomerulosclerosis index of each animal was determined by light microscopy at 400× magnification. Tubulointerstitial damage scores were evaluated at 100× magnification as follows: score 0: no change; score 1–5 depending on criteria met in given field of view: tubular dilatation, tubular atrophy, hyalin in tubular lumen, interstitial infiltration of mononuclear cells, interstitial fibrosis. 

Immunofluorescent staining of paraffin-embedded sections for SQSTM1 (p62) was carried out as described previously [10], with the use of citrate buffer pH 6.0 for heat-induced antigen retrieval and overnight incubation with the primary antibody then with Alexa Fluor-594-conjugated anti-rabbit IgG (Supplementary Table S1). The SQSTM1-positive stained area at magnification of 400× was evaluated for each slide using ImageJ v1.53 software (NIH, Bethesda, MD, USA).

### 4.4. Quantitative RT-PCR Analysis

Total RNA was extracted from mouse kidneys/primary cells using Trizol reagent (Invitrogen, Thermo, Waltham, MA, USA) according to the manufacturer’s instructions. RNA concentration and purity was assessed on a NanoDrop 1000 spectrophotometer (Thermo). Reverse transcription of RNA samples (1 µg per kidney and 500 ng per PTEC) into cDNA was performed using a high-capacity cDNA kit (Applied Biosystems/Life Technologies, Carlsbad, CA, USA). SensiFast SYBR Green I-based real-time PCR (Bioline, Meridian Bioscience, Memphis, TN, USA) was performed on a Bio-Rad CFX96 thermal cycler (Bio-Rad Hungary, Budapest, Hungary) with specific primer pairs (see Supplementary Table S2). The specificity and effectivity of PCR reactions were verified by melting curve analysis. Target gene and miRNA expression was normalized to 18S rRNA or U6 snRNA expression, respectively, using the 2^−ΔΔCt^ formula and expressed as fold expression relative to a calibrator sample. PCR experiments were performed in duplicates. Each gene expression is presented as mean ± standard deviation (SD). In each PCR experiment, appropriate positive and negative controls were included to verify the results, and to avoid false positive signals due to contamination.

### 4.5. Immunoblot Analysis

Immunoblots were performed as described previously [10]. Briefly, 3 mm^3^ kidney cortex or scraped PTEC samples were homogenized in ice cold RIPA lysis buffer. The protein concentration was measured using the BCA assay (Thermo Scientific, Waltham, MA, USA). Equal protein amounts (20 μg PTEC or 60 μg cortex) were loaded with 2× Laemmli buffer (Bio-Rad) on SDS-polyacrylamide gels. After separation, proteins were transferred on a nitrocellulose membrane by wet method. Transferred membranes were then blocked with 5% skim milk in TBST buffer and incubated with primary antibodies (see Supplementary Table S1) overnight at 4 °C. After serial washes in TBST, membranes were incubated with the appropriate HRP-conjugated secondary antibodies for 1 h at room temperature, as blots were visualized with the enhanced chemiluminescence (ECL) detection kit (Thermo). Tubulin or glyceraldehyde-3-phosphate dehydrogenase (GAPDH) were used as loading controls. Image Studio Lite 5.2 (Li-Cor Biosciences, Lincoln, NE, USA) software was used to examine and evaluate data.

### 4.6. Immunocytochemistry

Primary tubular epithelial cells at a density of 3 × 10^4^ cells per well were grown on gelatin-coated glass coverslips in 24-well plates. Cells were fixed in methanol (−20 °C) for 15 min on ice and permeabilized and blocked using 0.25% Triton-X (for 10 min) and 2% (*w*/*v*) donkey serum (for 30 min) in phosphate-buffered saline (PBS). Cells were incubated overnight at 4 °C with primary antibodies (see Supplementary Table S1). Next, the cells were incubated with a secondary antibody at room temperature for 1 h in the dark. Coverslips were transferred onto microscope slides with VectaShield mounting medium (Vector Laboratories, Newark, CA, USA) containing DAPI for nuclear staining.

Immunostaining reactivity of the cells for EGR2 and VIM was evaluated in a single-blinded fashion (unaware of the treatment groups) by calculating the average signal intensity of each stained area using ImageJ v1.53 and expressed as arbitrary units (a.u.). SQSTM1 staining was evaluated by calculating the percentage of positive cytoplasmic area with ImageJ.

## 5. Conclusions

**Figure 9:**
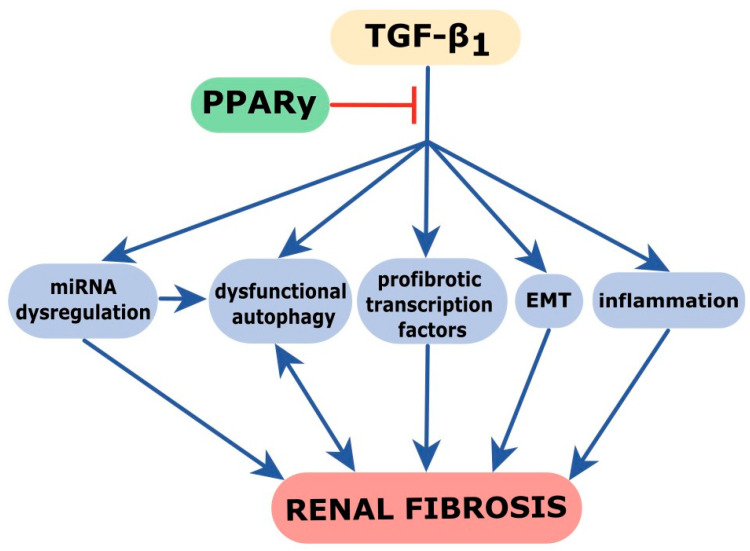
The proposed reno-protective effects of pioglitazone treatment in TGF-β-induced kidney fibrosis. Based on our in vitro and in vivo studies, we postulate that PPARγ might effectively counteract the TGF-β-induced miRNA dysregulation, autophagy dysfunction, pro-fibrotic transcription factor over-production, epithelial-to-mesenchymal transition (EMT), and inflammatory response of renal tubular epithelial cells. These multiple actions of PPARγ enhance its anti-fibrotic effect.

Taken together, we propose that pioglitazone treatment can rescue the dysfunctional autophagic activity in TGF-β-induced renal fibrosis and facilitates the clearance of damaged cellular organelles, restoring cellular function. This effect, together with the decreased expression of fibromiRs and pro-inflammatory molecules (Figure 9), likely account for the potential anti-fibrotic, anti-inflammatory, and protective effects of PPARγ agonists in chronic kidney disease, which are highly relevant for the according clinical therapy.

## Data Availability

Experimental data are available upon reasonable request to the corresponding author.

## Supplementary Materials

The following supporting information can be downloaded at: https://www.mdpi.com/article/10.3390/ijms242115520/s1.
