# Supplementary material for: Pioglitazone Protects Tubular Epithelial Cells during Kidney Fibrosis by Attenuating miRNA Dysregulation and Autophagy Dysfunction Induced by TGF-β
Source: Int J Mol Sci. 2023 Oct 24;24(21):15520. doi: 10.3390/ijms242115520 (PMC10649561; doi:10.3390/ijms242115520)
Supplement: Supplementary file 1 [file ijms-24-15520-s001.zip › ijms-2673968-supplementary.pdf]

Pioglitazone protects tubular epithelial cells during kidney fibrosis by attenuating miRNA dysregulation and autophagy dysfunction induced by TGF-β

Authors

Anna Manzéger[1,2], Gantsetseg Garmaa[1], Miklós M. Mózes[1,2], Georg Hansmann[3], Gábor Kökény[1,2]*

Affiliations

[1] Institute of Translational Medicine, Semmelweis University, 1089 Budapest, Nagyvárad tér 4, Hungary

[2] International Nephrology Research and Training Center, Semmelweis University, 1089 Budapest, Nagyvárad tér 4, Hungary

[3] Department of Pediatric Cardiology and Critical Care, Hannover Medical School, Hannover, Germany

Supplementary information

2 supplementary tables

Table S1.

**Antibody type**	**Dilution**	**Manufacturer**
Alexafluor-594-conjugated anti-rabbit IgG; #8890	1:200	Jackson Immunoresearch, Ely, UK
HRP-conjugated anti-mouse IgG; #7076	1:2000	Cell Signaling, Danvers, MA, USA
HRP-conjugated anti-rabbit IgG; #7074	1:2000	Cell Signaling, Danvers, MA, USA
mouse monoclonal anti-Gapdh; #MAB374	1:10000	Sigma-Aldrich, St. Louis, MO, USA
mouse monoclonal anti-tubulin	1:10000	Sigma-Aldrich, St. Louis, MO, USA
rabbit monoclonal anti-SQSTM1/p62; #39749	WB: 1:1000<br>ICC: 1:300	CellSignaling, Danvers, MA, USA
rabbit monoclonal anti-vimentin; #5741	1:500	Cell Signaling, Danvers, MA, USA
rabbit polyclonal anti-EGR2; #NB100-92327	WB: 1:2000<br>ICC: 1:300	Novus Biologicals, Centennia, USA
rabbit polyclonal anti-LC3A/B; #4108	WB: 1:1000	Cell Signaling, Danvers, MA, USA
rabbit polyclonal anti-TGF-β1; #3711	WB: 1:1000	CellSignaling, Danvers, MA, USA
rabbit monoclonal anti-EGR1; #4153	WB: 1:2000	CellSignaling, Danvers, MA, USA
rabbit monoclonal anti-P-Stat3; #9145	WB: 1:2000	CellSignaling, Danvers, MA, USA
rabbit monoclonal anti-Stat3; #4904	WB: 1:2000	CellSignaling, Danvers, MA, USA

Legend. Primary and secondary antibodies used for immunostaining and immunoblotting.

Table S2.

**Gene symbol**	**Forward primer**	**Reverse primer**
18S	TGGTTGCAAAGCTGAAACTTAAAG	AGTCAAATTAAGCCGCAGGC
Acta2	ACATAGCTGGAGCAGCGTCT	CCCACCCAGAGTGGAGAA
C3	TCCTTCACTATGGGACCAGC	TGGGAGTAATGATGGAATACATGG
Ccl2	TCCACCACTATGCAGGTCTC	GGGCATTAACTGCATCTGGCT
Clu	CTTAAGAGAAGGTGAAGATGAC	CAGGATTGTTGGTTGAACAG
Col1A1	CGTATCACCAAACTCAGAAG	GAAGCAAAGTTTCCTCCAAG
Ctgf	CCCGAGTTACCAATGACAATAC	CTTAGCCCTGTATGTCTTCAC
Eg2	TGACCAGATGAACGGAGTGG	ACTCGGATACGGGAGATCCA
Gapdh	CCATGACAACTTTGGCATTG	CCTGCTTCACCACCTTCTTG
Il6	GGAGCCCACCAAGAACGATAG	GTGAAGTAGGGAAGGCCGTG
Lc3b	GCTCATCAAGATAATCAGACG	GCATAAACCATGTACAGGAAG
Lgals3	GAAAAGAGTACTAGAAGCGG	CATTTTCCTGATTAGTGCTCC
Mmp2	GGACAAGAACCAGATCACATAC	CGTCGCTCCATACTTTTAAGG
Pparg	TTCGCTGATGCACTGCCTAT	GGAATGCGAGTGGTCTTCCA
Runx1	CGGTAGAGGCAAGAGCTTCA	GATGTCTTCGGGGTTCTC
Sqstm1	AATGTGATCTGTGATGGTTG	GAGAGAAGCTATCAGAGAGG
Tgfb1	TGGAGCAACATGTGGAACTC	CAGCAGCCGGTTACCAAG
Timp2	CAGGAAAGGCAGAAGGAGATG	GATCATGGGACAGCGAGTG

Legend. Primer sequences (5’ – 3’) used for qPCR of murine samples.
